# Toxicity of airborne particles—established evidence, knowledge gaps and emerging areas of importance

**DOI:** 10.1098/rsta.2019.0322

**Published:** 2020-09-28

**Authors:** Frank J. Kelly, Julia C. Fussell

**Affiliations:** NIHR Health Protection Research Unit in Environmental Exposures and Health, School of Public Health, Imperial College London, Sir Michael Uren Building, White City Campus, 80-92 Wood Lane, London W12 0BZ, UK

**Keywords:** particulate matter, toxicology, epidemiology, non-exhaust, microplastics, metabolomics

## Abstract

Epidemiological research has taught us a great deal about the health effects of airborne particulate matter (PM), particularly cardiorespiratory effects of combustion-related particles. This has been matched by toxicological research to define underlying mechanistic pathways. To keep abreast of the substantial challenges that air pollution continues to throw at us requires yet more strides to be achieved. For example, being aware of the most toxic components/sources and having a definitive idea of the range of associated disease outcomes. This review discusses approaches designed to close some of these knowledge gaps. These include a focus on particles arising from non-exhaust PM at the roadside and microplastics—both of which are becoming more relevant in the light of a shift in PM composition in response to global pressure to reduce combustion emissions. The application of hypothesis-free approaches in both mechanistic studies and epidemiology in unveiling unexpected relationships and generating novel insights is also discussed. Previous work, strengthening the evidence for both the adverse effects *and* benefits of intervention tell us that the sooner we act to close knowledge gaps, increase awareness and develop creative solutions, the sooner we can reduce the public health burden attributable to these complex and insidious environmental pollutants.

This article is part of a discussion meeting issue ‘Air quality, past present and future’.

## 1. Introduction—established evidence

### (a) Epidemiology

Particulate air pollution has been the focus of a global research effort for several decades. The aims have been not only to understand and describe associations between exposure and capability to adversely affect human health, but also to identify the plausible biological mechanisms that could explain and support these associations. The exceptional achievements began with the seminal epidemiological studies in the 1990s showing a clear association between increased respiratory and cardiovascular mortality and acute and chronic exposures to ambient particulate air pollution [1,2]. These findings have subsequently been substantiated in epidemiological studies conducted outside of the USA [3] and numerous attempts have followed to quantify the global annual burden of mortality due to particulate matter (PM) less than 2.5 µm in diameter (PM_2.5_). The current estimates approach 9 million [4]. Epidemiological investigations have also successfully delineated associations of particulate air pollution exposure with increases in respiratory and cardiovascular morbidity [5]. Evidence is particularly strong for reduced lung function, heightened severity of symptoms in individuals with asthma and chronic obstructive pulmonary disease [6] and ischaemic heart disease [6,7] and, in 2012, particulates in diesel fumes were classified as carcinogenic [8]. Data also link exposure with atherosclerosis [9] and a host of childhood respiratory conditions including an increased susceptibility to infection [10] and symptoms of low lung function [11].

### (b) Toxicology

The epidemiological work has been matched by a considerable toxicological research effort to define the underlying mechanistic pathways of toxicity elicited by airborne PM, and again the lungs and cardiovascular system have been particularly well studied. One such successful approach to investigate effects on the airways exposed human volunteers (healthy and/or mildly asthmatic) for 1–2 h to whole diesel exhaust (DE; particulates plus the associated gas phase) from an idling engine at concentrations ranging from environmentally relevant (PM with a diameter less than 10 µm [PM_10_] 100 µg m^−3^, nitrogen dioxide [NO_2_] 0.7 ppm) to those occasionally experienced in exceptionally busy diesel-dominated traffic environments (PM_10_ 300 µg m^−2^, NO_2_ 1.6 ppm). By performing blood, bronchoalveloar lavage and bronchial mucosal biopsy sampling after exposure, these studies have been instrumental in uncovering systemic and pulmonary inflammatory cascades following the stimulation of antioxidant defences and redox-sensitive signalling pathways [12–18]. Exposure to NO_2_ alone, at similar or higher concentrations for a longer duration, failed to elicit adhesion molecule upregulation or significant changes in inflammatory cells in the bronchial mucosa sampled, suggesting that the PM content of DE was the responsible pollutant [19]. In addition to the large number of studies on the inflammatory effects on airway epithelium and immune cells, a capacity of DE particles (DEP) to directly interact with airway nerve fibres responsible for respiratory symptoms has also been demonstrated [20].

In 2010, Brook and colleagues presented persuasive evidence that oxidative stress is also a critically important cause and consequence of PM-mediated cardiovascular effects. The latter are manifested through several, likely overlapping, pathways including at the functional level, endothelial dysfunction, atherosclerosis, pro-coagulant changes, alterations in autonomic nervous system balance and changes in blood pressure [7]. At the molecular level, principal pathways include (i) the instigation of pulmonary and systemic inflammation [21], (ii) the translocation of ultrafine and nanosize particles and/or particle constituents (organic compounds, metals) across the alveolar membrane into the systemic circulation possibly enabling interaction and localized toxicity within the vascular endothelium and/or cardiac tissue [22], and (iii) the activation of airway-sensitive receptors or nerves and subsequent autonomic nervous system imbalance [23]. At numerous points within each of these functional and molecular pathways there is potential for cellular oxidative imbalances to occur, as has been demonstrated in human experimental studies, healthy and diseased animal models, isolated organs and cell cultures [24].

### (c) Knowledge gaps

The past decades of epidemiological and toxicological research have taught us a great deal about the health effects of PM and particularly, the cardiorespiratory effects of roadside PM (primarily DEPs). Knowledge is, however, still lacking in many areas, two of which are discussed in this brief review. First, the differential toxicity of airborne PM, particularly in the light of the likely shift in composition in response to global pressure to reduce combustion emissions, and second what appears to be a growing range of disease outcomes that may ultimately be associated with exposure to airborne particulates.

## 2. Differential toxicity of ambient PM

### (a) The relevance

Health studies describing robust associations between ambient PM and ill health have contributed to the World Health Organization Air Quality Guidelines (WHO AQG) and national air quality standards that, owing to the technical limitations and costs of stationary monitoring networks, use the mass concentration of PM_2.5_ or PM_10_ as the metric. As a consequence, all particles are treated as equally toxic, without regard to their source and chemical composition. It is unlikely, however, that every component within the overall ambient PM mix is equally harmful to the exposed population. There has, therefore, been an enormous drive to identify which component(s)/source(s) of ambient PM, and/or which of their physical and chemical characteristic(s) are most harmful to health to facilitate reappraisal of air quality guidelines/standards and prioritize targeted PM control strategies to more effectively protect public health. Epidemiological and toxicological research findings have indeed shown that PM mass comprises fractions and sources with varying types and degrees of health effects but, despite this, the question of differential PM toxicity represents one of the most challenging areas of environmental health research [25].

### (b) The challenge

Rather than constituting a single entity, ambient particulate pollution is a complex, heterogeneous mixture that can exist in the atmosphere as solids or liquids. Primary PM is directly emitted from source, while secondary particles are formed following chemical reactions with other pollutants. The mix includes emissions from man-made activities as well as natural sources.

The organic particulate mix is particularly complex and constitutes around 10^4^–10^5^ different compounds in today's atmosphere [26]. These materials can be classified in several different ways: with respect to volatility, i.e. volatile organic compounds, semi-volatile organic compounds or condensed-phase compounds; primary or secondary condensed-phase organic compounds; carbonaceous particulate materials existing in the elemental carbon or organic carbon fraction. Since the latter is comprised of a very large number of individual compounds, observations of epidemiological associations unfortunately do not tell us about the identity and source(s) of the individual compound(s) that may be driving health effects. The major anthropogenic sources of organic materials in the atmosphere include internal combustion engines, wood and biomass burning, fuel oil combustion, natural gas combustion, biogenic emissions, resuspended road dust, tyre and brake wear and cooking emissions.

Airborne particles vary in chemical composition (and hence solubility and reactivity), mass, size, number, shape and surface area depending upon source and atmospheric processing. All of these properties have the potential to influence health effects. For example, with respect to size, particles can vary from a few nanometres to tens of micrometres. Particles with an aerodynamic diameter smaller than 0.1 µm (PM_0.1_), PM_2.5_ and between 2.5 and 10 µm (PM_10-2.5_) are termed ultrafine (UFPs), fine and coarse particles, respectively. The smaller particles, particularly UFPs, have a greater capacity to (i) penetrate the lung and probably translocate to extrapulmonary sites and (ii) adsorb toxic chemicals owing to a larger surface area to volume ratio. UFPs are, however, challenging to study in epidemiological settings that rely on central site measurements owing to their high spatial variability and high correlation with other combustion-related pollutants. Toxicological studies can also be challenging because of the rapid agglomeration of such particles. Attempts to identify specific effects of components/sources are complicated further since PM can vary in space and time as a consequence of atmospheric chemistry and weather conditions, as well as complex interactions with gaseous air pollutants (e.g. ozone and NO_2_) that share biologically plausible associations with various health endpoints.

### (c) The overall consensus

Unsurprising, the current database of experimental and epidemiological studies does not allow individual PM characteristics or sources to be definitely identified as being closely related to specific health effects [5,27,28]. It appears that the strengths of associations between effects and individual chemical components of the ambient aerosol vary from effect to effect and that the situation is further complicated by components being associated with certain effects in some locations, but not in others. The ambitious US NPACT studies—that used coordinated toxicology, epidemiology and exposure research to examine and compare the toxicity of PM components, gases and sources—concluded that ‘the studies do not provide compelling evidence that any specific source, component or size class of PM may be excluded as a possible contributor to PM toxicity’ [28]. In the context of organic PM, the findings of other large, multi-year large toxicological and epidemiological research programmes including SPHERES (Secondary Particle Health Effects Research), NERC (National Environmental Respiratory Center), ACES (Advanced Collaborative Emissions Study) and TERESA (Toxicological Evaluation of Realistic Emissions of Source Aerosols) have recently been reviewed [29]. Despite clear health impacts from emissions containing carbon-containing PM, difficulty remains in apportioning responses to certain groupings of carbonaceous materials, such as organic and elemental carbon, condensed and gas phases, and primary and secondary material. Another illustration of the variety of results reported in the literature is a systematic review of the findings of epidemiology, controlled human exposure and toxicology studies that used apportionment methods to relate sources of PM with human health outcomes [30]. Among the 29 studies reviewed, soil, sea salt, secondary sulphate, motor vehicle emissions, coal burning, wood smoke, biomass combustion, Cu smelter emissions, residual oil combustion and incinerator emissions were found to be associated with health outcomes. Another noteworthy quote on the subject from Krall and colleagues states ‘Associations with a given PM_2.5_ chemical component should be considered as potentially indicative of associations with another component or set of components with similar sources' [31]. It is in fact a belief of various commentators in the field that the literature suggests that various complex mixtures may be involved, and that the capability of PM to induce disease may be the result of multiple components acting through different physiological mechanisms [32,33]. Efforts continue to tease out which components and sources of PM are most harmful since to identify regulation targets can better protect human health. Epidemiological studies of sources are, however, challenging since source-specific exposures (e.g. PM from road transport) often are not directly measured and must be estimated by applying source apportionment models [34–36]. Using data from the Atlanta Commuting Exposure studies, Krall *et al.* found that exposures related to crustal and secondary pollution were associated with decreased lung function among asthmatic commuters [37]. Findings from another recent study that adopted source-apportioned PM_2.5_ concentrations suggest a role for emissions from spark ignition, and diesel vehicles, tyre and brake wear, residual oil combustion emissions from large building heating and nitrate particles in the triggering of acute cardiovascular events [38].

## 3. Particles of increasing interest and importance

### (a) Non-exhaust PM from road transport

**Figure 1.**
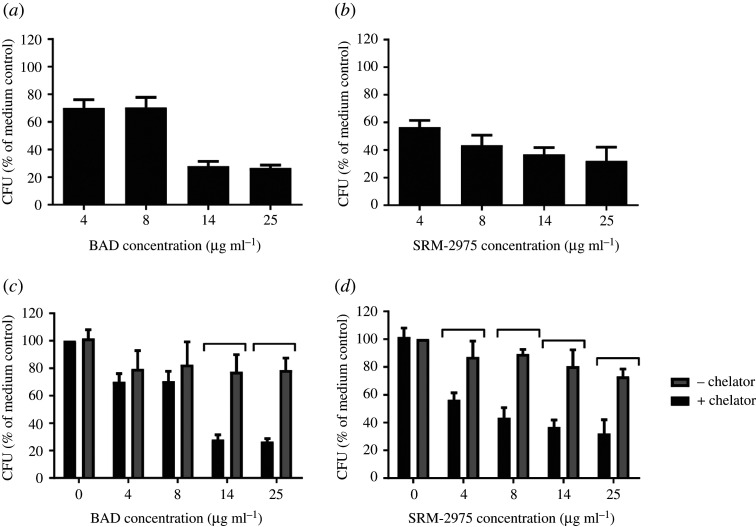
Quantities of *S. aureus* ingested by U937 cells over a 2 h period subsequent to 24 h incubation with (*a*) BAD, (*b*) DEP (SRM-2975), (*c*) BAD (±METAL CHELATOR DFO) and (*d*) DEP (SRM-2975) (±DFO). BAD, brake abrasion dust; CFU, colony-forming units; DFO, desferroxamine mesylate; SRM-2975, standard reference material 2975. [39]. Published by The Royal Society of Chemistry.

A recent toxicological study incorporating comparative data from different road traffic sources focused on brake abrasion dust (BAD) and DEPs [39]. This is highly relevant in that while there is an extensive literature on the health effects of engine emissions [40], the toxicity of non-exhaust PM—from brake wear, tyre wear, road surface wear and resuspended road dust—has not been extensively studied despite becoming a significant component of urban air pollution. Furthermore, this currently unregulated component of traffic emissions is expected to become proportionately more important, as vehicle exhaust PM emissions from road transport are expected to decrease over the coming years. The *in vitro* study by Selley and co-workers compared the relative toxicity that BAD and DEPs exert on airway macrophages to investigate whether the marked compositional difference between these particle species is reflected in their ability to perturb cell function [39]. Although BAD contained considerably more metals/metalloids than DEP, similar toxicological profiles were observed in U937 monocyte-derived macrophages at minimally cytotoxic doses (4–25 µg ml^−1^; 24 h). Responses to the particles included transient, dose- and metal-dependent increases in secretion of IL-8, IL-10 and TNF-α and decreased phagocytosis of *Staphylococcus aureus* and, for both particles, the metal chelation restored bacterial uptake to levels comparable with the particle-free control (figure 1).

### (b) Microplastics

**Figure 2.**
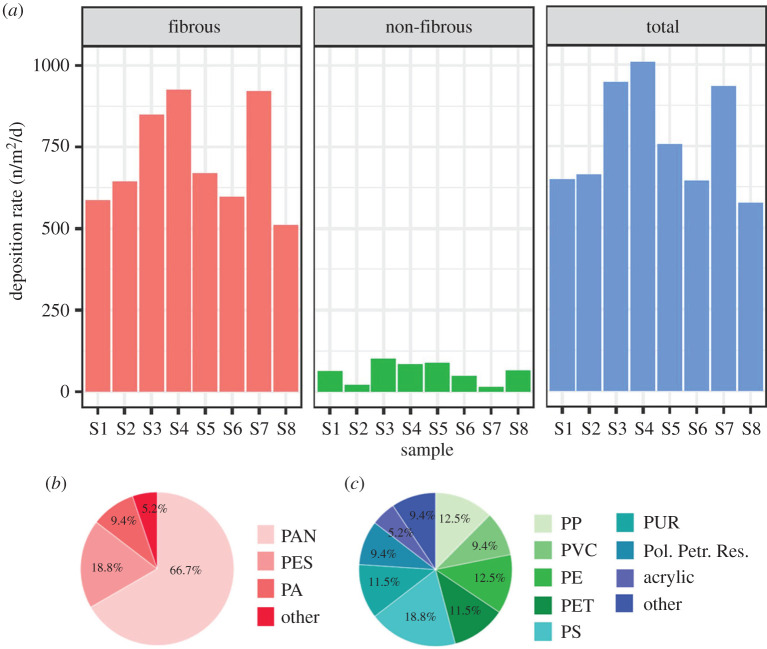
Time-series of deposition rates (n/m^2^/d) for fibrous, non-fibrous and total microplastics (*a*); proportional distribution of identified petro-chemical-based fibrous microplastics (*b*); proportional distribution of identified petro-chemical-based non-fibrous microplastics (*c*). PAN, polyacrylonitrile; PES, polyester; PA, polyamide; PP, polypropylene; PVC, polyvinylchloride; PE, polyethylene; PET, polyethylene terephthalate; PS, polystyrene; PUR, polyurethane; Pol. Petr. Res, polymerized petroleum resin [48]. (Online version in colour.)

Microplastics (less than or equal to 5 µm particles and fibres produced from the breakdown of larger items such as clothing, car tyres and mismanaged urban waste) are another particulate pollutant of increasing environmental concern owing to the astonishing global mass production of plastic plus its persistent nature in the environment [42] and synthetic physiological fluids [43]. While occurrence, sources and fate of airborne microplastics are still poorly documented, in part owing to the technological challenge associated with detection and identification, progress is being made in assessing atmospheric deposition in both indoor [44] and outdoor [45–48] environments. Of the latter, airborne microplastics have been measured in the major population centres of Paris [45], Dongguan [46] and London [48] as well as at a remote and pristine site in the French Pyrenees [47]. In central London, microplastics of various shapes, but primarily fibrous, were detected in all samples tested, with deposition rates ranging from 575 to 1008 per m^2^ per day. Across all samples, 15 different petrochemical-based polymers were identified (figure 2).

Evidence of a novel component of air pollution is, therefore, emerging while complementary existing fields indicate potential hazards. For example, evidence exists that people working in the textile industry experience respiratory symptoms and develop interstitial lung disease following exposure to nylon flock [49] and similar symptoms have been described among workers in facilities manufacturing polyethylene and polypropylene flock [50,51]. Although the toxicology of microplastics is an emerging field, several potential mechanisms exist through which harm to human health could occur. These may include the activation of pathways in response to the particle/fibre *per se* and/or leaching of adhered chemicals (owing to the surface area:volume ratio of microplastics, plus their surface hydrophobicity), from both additives incorporated during manufacture and contaminants accumulated from the environment. For example, an acute inflammatory response has been demonstrated in the lungs of rats after intratracheal instillation of nylon fibres of a respirable size (2 µm diameter, 14 µm length on average) [52]. Furthermore, inflammation induced by granular and spherical particles (polyethylene/polyethylene terephthalate) from abraded plastic prosthetic implants has also been reported [53,54]. With respect to the leaching of plastic-derived chemicals, potentially with reproductive, carcinogenic and mutagenic effects, there is no information on human tissues but transfer of plastic-derived chemicals from ingested waste to the tissues of marine-based organisms has been described [55].

## 4. Applications of omics approaches to explore mechanisms of toxicity

Much of our understanding of the mechanisms by which particulate air pollution elicits ill health has been gleaned from traditional, hypothesis-driven approaches that focus on *a priori* defined clinical parameters employing a limited number of endpoint assays [56,57]. These have been fundamental to epidemiologists in providing the means to support causal inference by providing associations between physiological endpoints (e.g. respiratory or cardiovascular symptoms) and underlying molecular events. The limitation lies in the inability to uncover the multiple molecular targets and novel pathways that are undoubtedly behind the toxicological response to complex environmental exposures.

**Figure 3.**
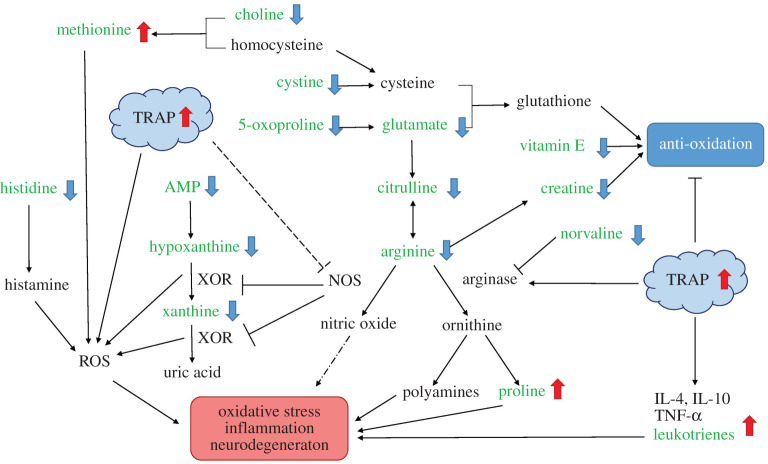
Potential molecular mechanisms underlying the effects of TRAP toxicity on individuals with asthma elucidated using untargeted high resolution metabolomics on the study participants. IL-4, interleukin 4; IL-10, interleukin 10; NOS, nitric oxide synthase; ROS, reactive oxygen species; TNF-α, tumour necrosis factor alpha; TRAP, traffic-related air pollution; XOR, xanthine oxidoreductase [69]. (Online version in colour.)

Current and more informative mechanistic studies, which heavily focus on questions rather than hypotheses, have a greater likelihood of unveiling unexpected relationships and generating novel insights that in turn can lead to hypothesis generation [58,59]. Advances in analytical technology, in the form of metabolomics, involves the simultaneous measurement, by mass spectrometry or nuclear magnetic resonance, of multiple small metabolites (less than 1 kDa) arising from specific cellular processes, such as energy production and storage, signal transduction and apoptosis [60]. Since metabolites are the terminal products of gene expression, i.e. the final consequence of biological function, their profiles in biological samples report on actual functional status. The staggeringly large amounts of information of such global analyses should not, however, be underestimated and gaining biologically relevant conclusions from a given metabolomics dataset requires a specialized data analysis. Notwithstanding such challenges, identifying metabolite perturbations caused by air pollution exposure is a particularly relevant and promising approach in characterizing the interactions of living organisms with their environment by identifying disregulated molecular pathways and predicting health endpoints [61]. It is not surprising, therefore, that to address issues such as differential toxicity, workers have adopted such an approach in both toxicological and epidemiological studies [62–68]. Experimental studies have investigated shifts in the metabolite profiles of bronchial wash (BW) and bronchoalveolar lavage (BAL) of healthy volunteers following exposure to biodiesel exhaust (BDE) compared with filtered air [62,63]. This approach greatly enhanced the number of metabolites that were detected and, in turn, novel pathways including alterations in energy metabolism and degradation of cell membrane lipids associated with BDE exposure. Notably, BDE-induced shifts in metabolite profiles of the BW versus BAL fluids differed appreciably, and a stronger response was detectable for peripheral regions of the lungs. Incorporation into epidemiological research has also been demonstrated to sensitively detect internal metabolic perturbations in healthy subjects, pregnant women and people with asthma following complicated exposures such as those present in urban environments [64,65,67–69]. A key finding from these studies has been the identification of several oxidative stress and inflammation-related pathways (including leukotriene, cytochrome P450, vitamin E, tyrosine, methionine and tryptophan metabolism) that were consistently associated with elevated pollution exposures. For example, in an analysis of healthy college students living close to a major urban highway, leukotriene, vitamin E, cytochrome P450 and methionine metabolic pathways were linked to longer-term (over 3 months) exposure to elevated traffic-related air pollution (TRAP), including black carbon (BC) and PM_2.5_ [65]. As illustrated in figure 3, Liang *et al*. [69] detected numerous significant metabolic perturbations associated with in-vehicle exposures during commuting, validated metabolites that were closely linked to several inflammatory and redox pathways and collectively implicated these mechanisms as part of the impact of TRAP toxicity in asthmatic individuals.

## 5. The wider threat to human health

Emerging epidemiological and experimental data from a growing number of studies suggest that particle exposure may exert a wider threat to human health, beyond the cardiorespiratory systems, by negatively influencing a broader number of diseases including adverse birth outcomes [71–73], slower rates of cognitive development in children [74,75] and accelerated cognitive decline in adults [76,77]. There remain substantial gaps in our knowledge as to possible causal pathways for these relatively new scientific observations. This could, however, be due to the translocation of the smallest particles in the overall mix into the target organs as well as more indirect pathways acting through inflammatory mediators produced in response to the particles.

Animal studies have documented the ability of small inhaled particles to reach the brain, with evidence suggesting that this occurs following deposition in either the nasal epithelium (via the olfactory nerve) or the alveolar epithelium by entering into the systemic circulation and eventually crossing the blood-brain barrier [78]. Importantly, the translocation of airborne particles to the brain is potentially supported by human evidence as Maher *et al*. reported the presence in postmortem brain samples of magnetite nanoparticles, consistent with those formed by combustion and/or friction-derived heating [79].

The numerous investigations into whether nanoparticles can cross the placenta show a dependency on size, shape and surface charge [80], while Valentino *et al*. [81] demonstrated ‘nanoparticle-like’ aggregates in the cytoplasm of placental trophoblastic cells of rabbits following exposure to aerosolized DEPs. These experimental data are also supported by human evidence with Bove *et al*. [82] reporting the presence of BC particles in placenta at both the maternal and fetal side. Such findings confirm the translocation of ambient PM directly to the fetus and represent a potentially novel mechanism to explain adverse health effects from early life onwards.

### (a) Hypothesis-free analysis

**Figure 4.**
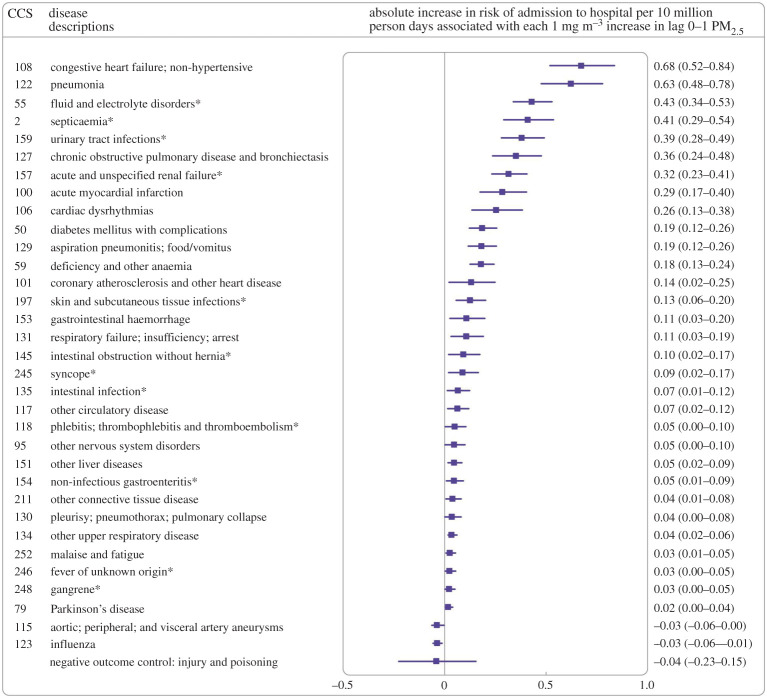
Analysis showing absolute increases in risk of hospital admission, ordered from highest to lowest, associated with each 1 µg m^−3^ increase in lag 0–1 PM_2.5_ [83]. CCS, Clinical Classification Software code. * Indicates newly identified disease groups. Reproduced from Short term exposure to fine particulate matter and hospital admission risks and costs in the Medicare population: time stratified, case crossover study, Wei *et al*. [83] with permission from BMJ Publishing Group Ltd. (Online version in colour.)

The quest to uncover associations between ambient PM and *all* possible diseases, including prevalent but rarely studied ones, has recently been tackled by using a hypothesis-free analysis of a large dataset [83]. This study analysed more than 95 million hospital admissions of Medicare beneficiaries plus PM_2.5_ concentrations on the day before presentation over 13 years. In addition to confirming previously established associations between short-term PM_2.5_ concentration and cardiorespiratory disease, diabetes mellitus and Parkinson's disease, the researchers found that each 1 µg m^−3^ increase in PM_2.5_ was associated with 2050 extra hospital admissions as a consequence of previously unassociated diseases (figure 4). The latter included fluid and electrolyte disorders, acute and unspecified renal failure, septicaemia, intestinal obstruction without hernia and urinary tract infections. Moreover, associations remained consistent when restricted to days when daily PM_2.5_ concentrations fell below the WHO 24 h AQG.

## 6. Discussion

Recent decades of epidemiological and toxicological research have taught us a great deal about the health effects and underlying toxicology of PM, particularly the cardiorespiratory effects of roadside, combustion-related particles. To keep abreast of the substantial challenges that air pollution continues to throw at us requires yet more strides to be achieved. The multi-disciplinary efforts to identify the most toxic components/sources and the range of associated disease outcomes must continue. Closure of some of today's knowledge gaps will be fundamental to inform the revision of air quality guidelines and standards, set effective mitigation strategies and assist their ultimate remit of protecting human health.

This is particularly relevant owing to predicted shifts in PM composition in response to global pressure to reduce combustion emissions. Particles arising from the non-exhaust component of traffic emissions are prime examples of current challenges on which to focus on. These have become a significant component of traffic-related PM and are projected to become a more dominant source. Added to this, particle characteristics with respect to both (small) size and (metallic) composition suggest that this currently unregulated source that is concentrated in highly populous urban environments may be a particularly hazardous one. While recent research suggests that the capacity of BAD to harm pulmonary cells is equivalent to that of DEP [39], unambiguous toxicological data are currently lacking. For example, for the most part, toxicological studies of tyre wear have employed pulverized or size-fractionated material, rather than that formed from ‘real world’ friction between tyres and roadways. In addition no direct epidemiological studies on non-exhaust PM at the roadside have been undertaken. New epidemiological and toxicological research, incorporating future trends (e.g. potential benefits of regenerative braking versus added risks associated with increased tyre and road wear from heavier electric/self-driving vehicles), should be undertaken to further understand the potential health risk of this aspect of vehicular pollution. Without such research, policy changes to control emission sources and benefit human health will be difficult.

The contribution of microplastics to the risks that airborne PM inflicts on human health is another timely research field that has emerged. Early work presenting evidence of airborne plastics in indoor air [44] and outdoors, in populous urban environments [45,46,48], raises concern for public health, especially with a predicted increase in plastic use, particularly in the textile sector, pointing to a proportional increment in airborne microplastic concentrations. A robust evidence base characterizing population exposure is, therefore, required. We also need to establish the toxic characteristics of microplastics, their behaviour in the body, and what, if any, constitutes a safe threshold for exposure when plastics are inhaled.

Environmental metabolomics has emerged as a means to provide a broad-spectrum of measurements of human metabolism that may reveal biological effects and associated toxicological mechanisms associated with an exposure to particulate air pollution. Detecting and monitoring markers of adverse outcomes following exposure to different air pollutants in large human cohorts is limited at present [84,85], but could divulge differential toxicities and thereby help to target regulatory efforts to those pollutants that pose the greatest risk to public health. Continued development of this field, in combination with complementary ‘omics’ technologies such as genomics and proteomics, as well as traditional hypothesis-led research will be crucial to help strengthen the causal basis for the epidemiological findings that associate air pollution with an ever growing number of diseases. Indeed, research that has used hypothesis-free analysis and predicted epidemiology via toxicology suggests that current figures for PM_2.5_-associated morbidity, which focus on established disease associations, might be considerable underestimates [83,86]. This again calls upon more epidemiological research to investigate newly reported associations, and particle toxicology to provide plausible biological mechanisms that could explain and support these associations.

As the burden of disease associated with particulate air pollution becomes more apparent, it is ever more clear that there is much still to learn. Previous work, strengthening the evidence for both the adverse effects *and* benefits of intervention [11,87,88] tell us that the sooner we act to close knowledge gaps, increase awareness and develop creative solutions, the sooner we can reduce the public health burden attributable to this complex and insidious environmental pollutant.

## Data accessibility

Data presented in this review can be accessed through previously published articles, a list of which is provided.
